# Study on repairing canine mandibular defect with porous Mg–Sr alloy combined with Mg–Sr alloy membrane

**DOI:** 10.1093/rb/rbz046

**Published:** 2020-01-30

**Authors:** Shanning Zhang, Xirao Sun, Chunyu Kang, Man Yang, Yuan Zhao, Chengyue Wang

**Affiliations:** 1 Department of Prosthodontics, Second Affiliated Hospital of Jinzhou Medical University, Jinzhou 121000, China; 2 Department of Prosthodontics, JinZhouShi Oral Cavity Hospital, Jinzhou 121000, China

**Keywords:** Mg**–**Sr alloy, Mg**–**Sr alloy membrane, mineralized collagen membrane, mandibular defects, guided bone regeneration

## Abstract

To discuss the feasibility of the application of porous Mg**–**Sr alloy combined with Mg**–**Sr alloy membrane in the repair of mandibular defects in dogs. The second and third mandibular premolars on both sides were extracted from six dogs. The model of mandible buccal fenestration bone defects were prepared after the sockets healed. Twelve bone defects were randomly divided into groups A and B, then Mg**–**Sr alloy was implanted in bone defects of group A and covered by Mg**–**Sr alloy membrane while Mg**–**Sr alloy was implanted in bone defects of group B and covered by mineralized collagen membrane. Bone defects observed on cone beam computed tomographic images and comparing the gray value of the two groups after 4, 8 and 12 weeks. After 12 weeks, the healing of bone defects were evaluated by gross observation, X-ray microscopes and histological observation of hard tissue. Bone defects in each group were repaired. At 8 and 12 weeks, the gray value of group A was higher than that of group B (*P *<* *0.05). At 12 weeks, the bone volume fraction of group A was higher than that of group B (*P *<* *0.05). The newly woven bone in group A is thick and arranged staggered, which was better than that of group B. Porous Mg**–**Sr alloy combined with Mg**–**Sr alloy membrane could further promote the repair of mandibular defects, and obtain good osteogenic effect.

## Introduction

Due to severe dental pulp disease, periodontal disease, trauma, surgery and congenital or anatomical conditions, mandibular defects are quite common and pose a substantial clinical and biomedical burden. Bone graft is an effective method for repairing bone defect.

Autogenous bone, bio-derived bone and synthetic bone graft substitute (nonmetallic material or metallic material) are often used to repair wide-bound mandibular defects. The ilium or tibia is often used because of limited bone mass. It is necessary to prepare another operation area, which increase the chance of infection and other complications, the operation time and cost [1]. The bio-derived bone carries the risk of spreading disease and immune rejection [2]. The outstanding problem of nonmetallic materials lies in their insufficient mechanical strength, which are the main reasons of its poor outcome after repair [3]. Metallic materials (titanium plate, titanium mesh, etc.) usually need to be removed by secondary surgery, which increases the pain of patients [4].

Mg and its alloys *in vivo* do not cause acute reaction and obvious inflammatory reaction. Due to their degradability, the ability to promote bone healing and biocompatible degradation/corrosion products, Mg and its alloys have shown potential as biodegradable metallic materials for orthopedic applications [5]. In our preliminary experiment, the magnesium-strontium alloy was used to repair the mandible bone defect in dogs, the results showed good osteogenic effect [6].

Guided bone regeneration (GBR) is often used to repair the small mandibular defects because of the capacity of bone regeneration. The previous study of our group found that using pure magnesium membrane to repair the vertical bone defect of canine mandible achieved a good effect. It shows good biocompatibility and safety. But pure magnesium degrades too quickly and does not match the process of new bone formation.

In recent years, the author’s research group has done a lot of research on magnesium-based metal materials *in vivo* and *in vitro*. On the basis of previous experimental research, in this experiment, porous magnesium-strontium alloy was used as bone graft material, combined with magnesium-strontium alloy barrier membrane, to explore the feasibility of repairing mandibular defects in dogs.

## Materials and methods

### Animals

This study investigated six hybrid dogs (approximately 12 months old and 12–15 kg in weight) that offered by the Animal Center of Jinzhou Medical University, Jinzhou, China. Adequate measures were taken to minimize the pain or discomfort to the experimental animals, and the experiments were conducted in accordance with the International Standards on Animal Welfare and the Ethical Standards of the Committee on Animal Experimentation of our institution.

### Materials

**Figure 1:**
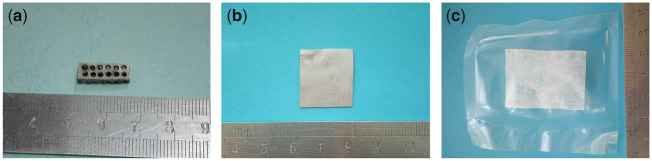
Microarc oxidation porous Mg–Sr alloy (**a**), Mg–Sr alloy membrane (**b**) and mineralized collagen membrane (**c**)

**Figure 2:**
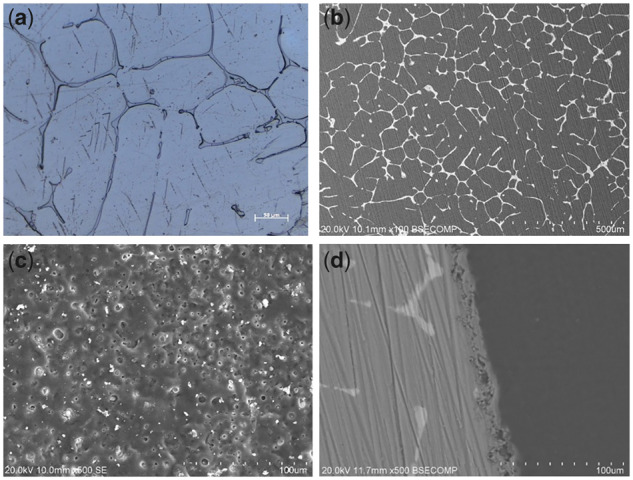
Optical micrograph (**a**) and back-scattered electron (BSE) images (**b**) of the as-cast Mg–Sr alloy; surface and cross-sectional morphologies of the microarc oxidation (MAO) coating (**c**, **d**) [8]

Microarc oxidation porous Mg**–**Sr alloy (Mg**–**1.5Sr in wt%, 15 mm × 5mm × 3mm) and Mg**–**Sr alloy membrane (Mg**–**1.5Sr in wt%, 20 mm × 20mm × 0.38 mm) were provided by Institute of Metal Research, Chinese Academy of Sciences. Mineralized collagen membrane (20 mm × 20mm × 0.38 mm) was produced by Beijing Allgens Medical Science & Technology Co., Ltd. This material has a three-dimensional pore-like structure similar to that of natural cancellous bone, which is a hydroxyapatite and type I collagen composites synthesized using *in vitro*: bilayer membrane structure, nano-hydroxyapatite (20–40%), porosity (75–90%) and aperture (50–500μm) [7] (Figs 1 and 2).

### Experimental procedures

**Figure 3:**
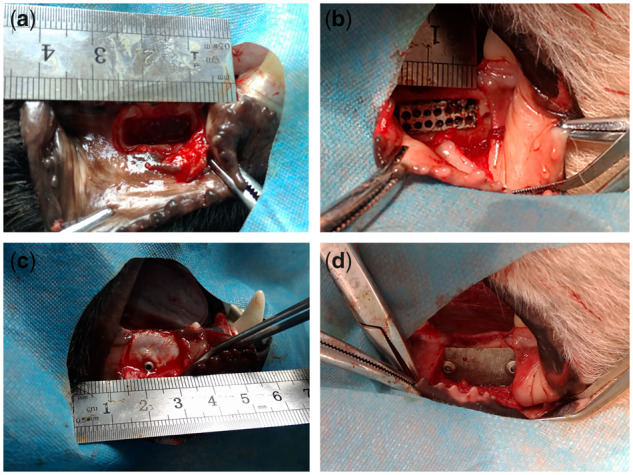
(**a**) The model of mandible buccal fenestration bone defect (15 mm × 5mm × 3mm); (**b**) implantation of Mg–Sr alloy; (**c**) mineralized collagen membrane; (**d**) Mg–Sr alloy membrane

**Figure 4:**
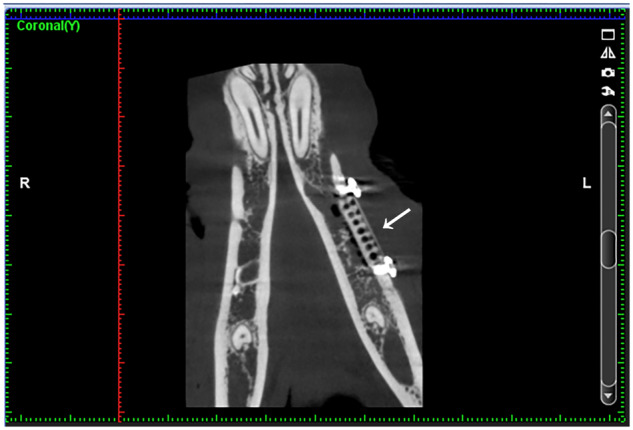
Postoperative CBCT imaging in experimental group. The implant material and metal barrier membrane can be clearly seen at the white arrow

Animals were anesthetized with xylazine hydrochloride injection (0.08–0.1 ml/kg). The second and third mandibular premolars on both sides were extracted from six dogs. The model of mandible buccal fenestration bone defects were prepared after the sockets healed. Trapezoid incision was made in the buccal gingival of the edentulous area to expose an operative area. The bone defect was made at 2 mm below the crest of the alveolar ridge. The edge of the defect was located by dental high-speed handpiece according to the designated depth (3 mm). Bone tissue was removed with osteotome and washed with normal saline to remove excess bone debris. Twelve bone defects were randomly divided into groups A and B. Mg**–**Sr alloy was implanted in bone defects of group A and covered by Mg**–**Sr alloy membrane while Mg**–**Sr alloy was implanted in bone defects of group B and covered by mineralized collagen membrane. The infection prophylaxis was provided with 4 wu/kg penicillin preoperatively and 3 days postoperatively. Part of the procedure is shown in Figs 3 and 4.

The general condition of the dogs was observed after the surgery, including diet, general activity, wound healing and the presence of infection. After 12 weeks, the condition of the bone defect area was observed *in vitro*.

**Figure 5:**
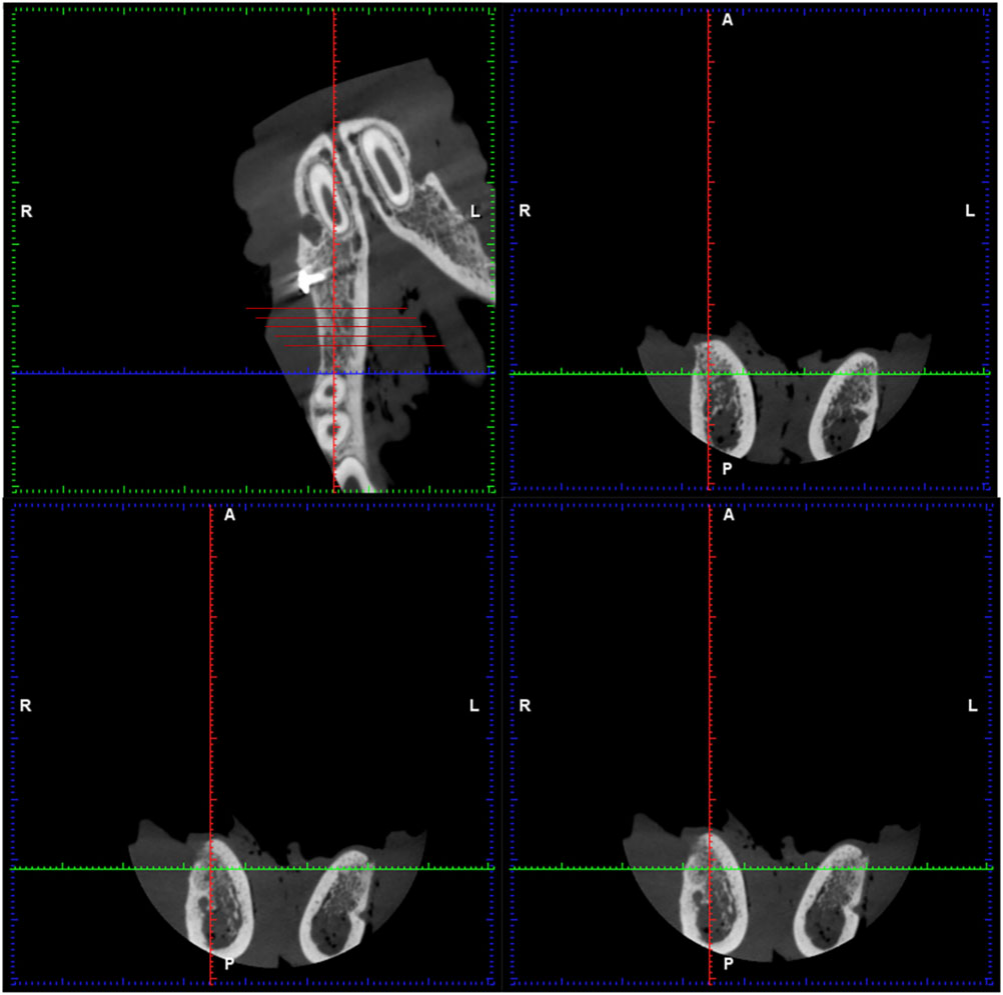
Measured by Planmeca Romexis viewer. The bone defect area is equally divided into five sections from distal to mesial, and the gray values of the new osseous tissue in the second, third and fourth section were calculated, respectively

After 4, 8 and 12 weeks, the bone defect areas were observed by CBCT (Planmeca, Finland). Three-dimensional measurement and positioning of image data were made by Romexis Viewer. According to the study of Ioku *et al.* [9], the bone defect area is equally divided into five sections from distal to mesial, and the gray values of the new osseous tissue in the second, third and fourth section were calculated, respectively (hounsfield unit, HU). In order to ensure that the data are accurate, each data were measured three times by the same person, and the average value is taken as the final data (Fig. 5).

At 12 weeks, the healing of bone defects were evaluated by X-ray microscope and histological observation of hard tissue. The ultrastructure of the mandibular defect area was analyzed by X-ray microscopy (Xradia Versa XRM-500, ZEISS, Germany). A region of interesting (1 mm × 1mm × 1mm) marked as ROI was selected in the central position of the new bone region, in which the bone volume fraction (bone volume/total volume, BV/TV) was calculated. Then, the bones of two groups were prepared into sections for histological observation and analysis.

### Data analysis

The data were analyzed with SPSS 18.0 statistical software (SPSS, US, IBM), and the repeated measurement analysis of variance/independent samples *T*-test was performed with a difference level of *P *<* *0.05 after the tests of normality and homogeneity of variance. Measurement data of the two groups were expressed as mean ± standard deviation.

## Results

After the operation, all the dogs were in good condition, and the wound had healed well without signs of infection.

The collecting dates of gray values were analyzed by repeated measurement analysis of variance as shown in Table 1. We found that at 8 and 12 weeks, the gray values of group A are higher than that of group B, and the difference was statistically significant (*P *<* *0.05).

**Table 1:** Measurement results of gray values (HU) (mean ± SD, *n* = 18)

	4 weeks	8 weeks	12 weeks
Group A	502.39 ± 33.06	623.83 ± 25.65	731.50 ± 29.45
Group B	495.56 ± 28.18	602.11 ± 29.46	703.72 ± 30.41

**Figure 6:**
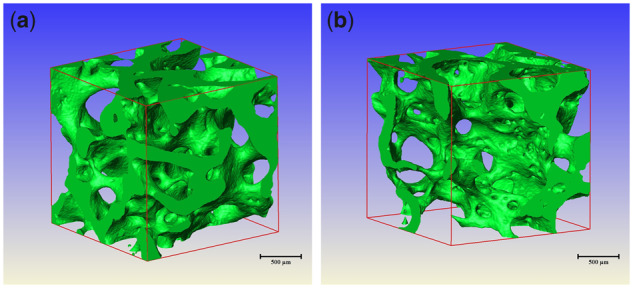
X-ray microscope images of VOI region in each group. (**a**) X-ray microscope images of VOI region in group A. (**b**) X-ray microscope images of VOI region in group B

At 12 weeks after the operation, we can see from the X-ray microscope images: images of Mg**–**Sr alloy cannot be seen at each group. The bone tissue section is loose and porous, the bone defect area is filled with new bone trabeculae and the bone trabeculae arranged loosely. The three-dimensional structure of bone trabeculae in group B is not as close as that of group A (Fig. 6). The collecting dates of bone volume fraction (BV/TV) in ROI were analyzed by independent samples *T*-test as shown in Table 2. The bone volume fraction of group A is higher than that of group B, and the difference was statistically significant (*P *<* *0.05).

**Table 2:** Measurement results of gray values (HU) (mean ± SD, *n* = 6)

	BV/TV
Group A	24.63 ± 1.71
Group B	21.82 ± 1.79

**Figure 7:**
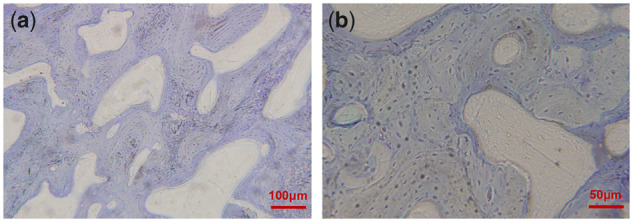
Bone hard tissue slicing in experimental area of group at 12 weeks (toluidine blue staining, (**a**) & (**b**): different magnification is represented by the scale in the figure)

**Figure 8:**
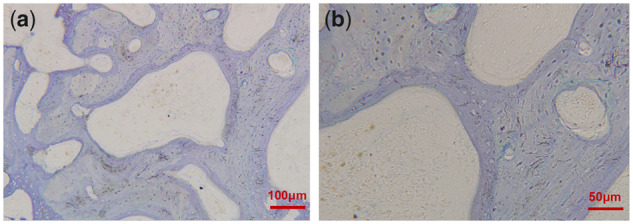
Bone hard tissue slicing in experimental area of group B at 12 weeks (toluidine blue staining, (**a**) & (**b**): different magnification is represented by the scale in the figure)

At 12 weeks after operation, the bone sections stained with toluidine blue were observed under light microscope: There were no Mg**–**Sr alloy materials in both groups, the new bone tissue was filled with defect area, the sections showed reticular porous structure, interlaced braided bones and the density of arrangement in group A was higher than that in group B (Figs 7 and 8).

## Discussion

The GBR technique used in this experiment has been proved to be a reliable technique for bone increment [10]. The GBR is to place a barrier membrane in the area of bone defect to form a relatively closed space to isolate the surrounding fibrous connective tissue while promoting the osteoblast to grow into the site of the bone defect, in order to achieve the goal of repairing bone defect [11]. Dimitriou *et al.* [12] pointed out that the application of barrier membrane is necessary in bone defect repair.

With the extensive clinical application of GBR technique, the success rate of implant surgery in bone defect area has been improved obviously. At present, GBR membranes include nonabsorbable membranes (Gore-Tex membrane and pure titanium membrane) and absorbable membranes (collagenous membrane and ester/lactide copolymer membrane). Titanium membrane has been widely reported in clinical application. It has the advantages of good osteogenic effect, but it also has the disadvantages of easy exposure and need to be removed. Sun *et al.* [13] showed that mineralized collagen membrane could play a synergistic role with bone graft material, slow down the absorption of alveolar bone and promote the formation of new bone. However, absorbable biomembrane may affect its osteogenic effect because of collapse.

As a degradable implant material, Mg and its alloy will not cause acute reaction and obvious inflammatory reaction after implantation, and the degradation of Mg and its alloys can also promote bone healing [14]. However, they degrade *in vivo* for a shorter time, usually less than 12 weeks longer than the time required for bone tissue healing [15]. Moreover, excessive H_2_ produced during degradation will affect the healing of bone tissue to some extent.

The corrosion resistance and bioactivity of Mg alloys can be improved by purification, alloying, improvement of processing technology and surface modification. Liu *et al.* [16] found that in 3% NaCl solution, the hydrogen evolution of high-purity Mg was significantly lower than that of ordinary pure Mg. However, the mechanical properties of pure Mg are not good enough to meet the requirements of clinical application [17].

Alloying is a more common method to improve the properties of the alloy. Strontium, which belongs to the second main group element, has similar chemical, metallurgical and biological functions as Mg [18]. Sr is an essential trace metal element for human, the recommended daily intake is about 2 mg. Content of Sr in human body is about 140 mg with 99% of the element being stored in bone.

Sr can improve the corrosion resistance of the alloy by improving its surface properties [19]. Frasnelli *et al.* [20] found that adding Sr to hydroxyapatite could significantly increase the activity of osteoblasts. Gu *et al.* [18] found that the mechanical properties and corrosion resistance of Mg**–**Sr alloy can be improved obviously when the Sr content in the alloy is less than 2 wt%.

It has been shown that microarc oxidation is an effective surface modification method and can improve the corrosion resistance of Mg alloy [21]. We have studied the surface modification of Mg**–**Sr alloy *in vitro*. We studied three commonly used coatings on Mg**–**Sr alloy, including microarc oxidation coating, electrodeposition coating and chemical conversion coating, and compared these coatings for requirements of favorable degradation and biological performances. The results indicate that the microarc oxidation coating on Mg**–**Sr alloy exhibited the best corrosion resistance and cell response among these coatings, and is proved to be more suitable for the orthopedic application [8].

Ge *et al.* [22] use the Mg**–**Sr alloy with the same composition as in this experiment to repair the rabbit radius obtained good osteogenic effect. At the same time, in the preliminary experiment of our group, the Mg**–**Sr alloy was used to repair the mandible bone defect of the dog, the results showed good osteogenic effect [6]. This experiment is also verified. In this experiment, the effect of osteogenesis was observed by using Mg**–**Sr alloy combined with Mg**–**Sr alloy membrane.

CBCT can provide higher image resolution, less radiation dose and exposure time than spiral CT, and is widely used in oral and maxillofacial imaging. It has been reported that there is a significant linear correlation between the gray value of CBCT and that of spiral CT [23]. The gray value of CBCT can be used to evaluate the bone mineral density. At 8 and 12 weeks, the gray value of group A was higher than that of group B, and the difference is statistically significant (*P *<* *0.05). The results showed that the bone mineral density of group A was higher than that of group B.

The bone volume fraction represents the percentage of trabecular volume to the total volume of cancellous bone in unit volume [24], which is the main marker for evaluating bone mass level. There was significant correlation between bone volume fraction obtained by X-ray microscope and gray value based on CBCT [25]. In this experiment, the bone volume fraction in group A was higher than that in group B, and the difference was statistically significant (*P *<* *0.05), suggesting that the trabeculae in group A were larger in volume and denser in bone, which was consistent with the results of CBCT.

It is reported that the regeneration of bone tissue defects requires at least 12 weeks [26]. The Mg**–**Sr alloy used in this experiment was completely degraded at the 12th week after operation, so it is necessary to further enhance the corrosion resistance of the Mg**–**Sr alloy in order to achieve a complete match between the degradation rate of the material and the process of bone defect repair and regeneration. Because all hard tissues (including osseous tissue) in the human body are composite structures, at present, the most promising biological bone graft materials are composite bone graft materials consisting of two or more than two kinds of materials, so as to make up for the shortcomings of single materials. Metal matrix composite based on Mg alloy is expected to be the research direction of optimizing corrosion resistance of Mg**–**Sr alloy.

## Conclusion

Mg**–**Sr alloy combined with Mg**–**Sr alloy membrane could further promote the repair of mandibular defects, and obtain good osteogenic effect. However, their degradation rate is still not exactly matched with the regeneration process of bone tissue. In the future, it is necessary to continuously adjust the corrosion resistance of Mg–Sr alloy in order to match the degradation rate of the material with the process of bone repair and regeneration.

## Funding

This work was supported by Science and Technology Fund of Liaoning Province (20180530071).

*Conflict of interest statement*. None declared.
